# Robotic-Assisted Adrenalectomy for Presumed Metastasis in a Male Patient With Breast Cancer: A Case Report

**DOI:** 10.7759/cureus.91583

**Published:** 2025-09-04

**Authors:** Rohan M Patel, Sydney Korsunsky, Galal El-Gazzaz, Alia Abdulla

**Affiliations:** 1 Dr. Kiran C. Patel College of Osteopathic Medicine, Nova Southeastern University, Fort Lauderdale, USA; 2 Department of Surgery, Broward Health Medical Center, Fort Lauderdale, USA; 3 Department of Breast Surgical Oncology, Broward Health Medical Center, Fort Lauderdale, USA

**Keywords:** adrenalectomy, adrenal incidentaloma, estrogen receptor-positive carcinoma, hypermetabolic adrenal lesion, invasive ductal carcinoma, male breast neoplasms, metastatic lesion, oncology, robotic surgery, surgical oncology

## Abstract

We report the case of a 57-year-old male with T1N1M0, estrogen receptor-positive (ER+), progesterone receptor-positive (PR+), and human epidermal growth factor receptor 2-negative (HER2-), corresponding to American Joint Committee on Cancer Stage IIA (AJCC Stage IIA) invasive ductal cell carcinoma of the left breast, who underwent a radical mastectomy with adjuvant chemotherapy. CT and MRI imaging demonstrated an adrenal nodule that persisted even after the patient's chemotherapy regimen was completed. Subsequent PET imaging detected a hypermetabolic left adrenal mass concerning for distant metastasis. Ultimately, the patient and care team decided that a robotic-assisted left adrenalectomy was likely the best course of action. Intraoperative frozen and final pathology both confirmed a benign adrenal fibroadenoma. This case highlights the diagnostic challenges faced when distinguishing adrenal metastasis from benign lesions in patients with a history of malignancy, especially when imaging findings are nonspecific. The case emphasized the sheer importance of multidisciplinary evaluation and potential surgical intervention in rare malignancies, such as male breast cancer, where existing guidelines are often extrapolated from female populations.

## Introduction

Male breast cancer is a rare malignancy, accounting for fewer than 1% of cumulative breast cancer cases and fewer than 1% of cancers in men [1]. Due to its diagnostic infrequency, it is often not a prominent differential diagnosis and is noticed later in the progressive course of the disease compared to that of females [2]. A radical mastectomy is the standard primary surgical intervention, typically combined with concomitant endocrine therapy, chemotherapy, or radiation, contingent upon staging and receptor status [3]. The most common sites of distant metastasis include the lungs, liver, and brain. Adrenal metastases, though possible, are rare and are infrequently the sole site of distant spread. When an adrenal mass is incidentally discovered in a patient with known malignancy, the clinical assumption tends to favor metastatic disease, especially in the absence of benign attributes [4]. Imaging alone, however, may be insufficient to distinguish between benign and malignant lesions [5]. Hormonal evaluation can be useful for ruling out primary adrenal lesions; concentrations of plasma metanephrines, catecholamines, renin, and aldosterone can be utilized in cases of suspected pheochromocytomas or Conn syndrome. The issue, however, is that many of these adrenal laboratory parameters are nonspecific and may be elevated in various somatic disease states [6]. For this reason, both the American College of Radiology and the European Society of Endocrinology recommend that an adrenal biopsy be performed. This procedure, however, comes with risks, including pneumothorax, infection, and tumor dissemination [7]. After persistent equivocal imaging findings and poor response to medical management, surgical oncology teams may advocate for a complete resection of the affected adrenal gland, considering a patient’s history of malignancy [8]. In this report, we present a rare case of a male breast cancer patient with an incidentally discovered left adrenal mass, presumed to represent solitary metastatic disease based on the enhancement seen on PET imaging. This instance illustrates the diagnostic dilemma of adrenal masses in cancer patients, particularly in rare populations, while highlighting the importance of thorough multidisciplinary evaluation, especially when there is little research in the field due to the diagnostic rarity of this case. 

## Case presentation

**Figure 1:**
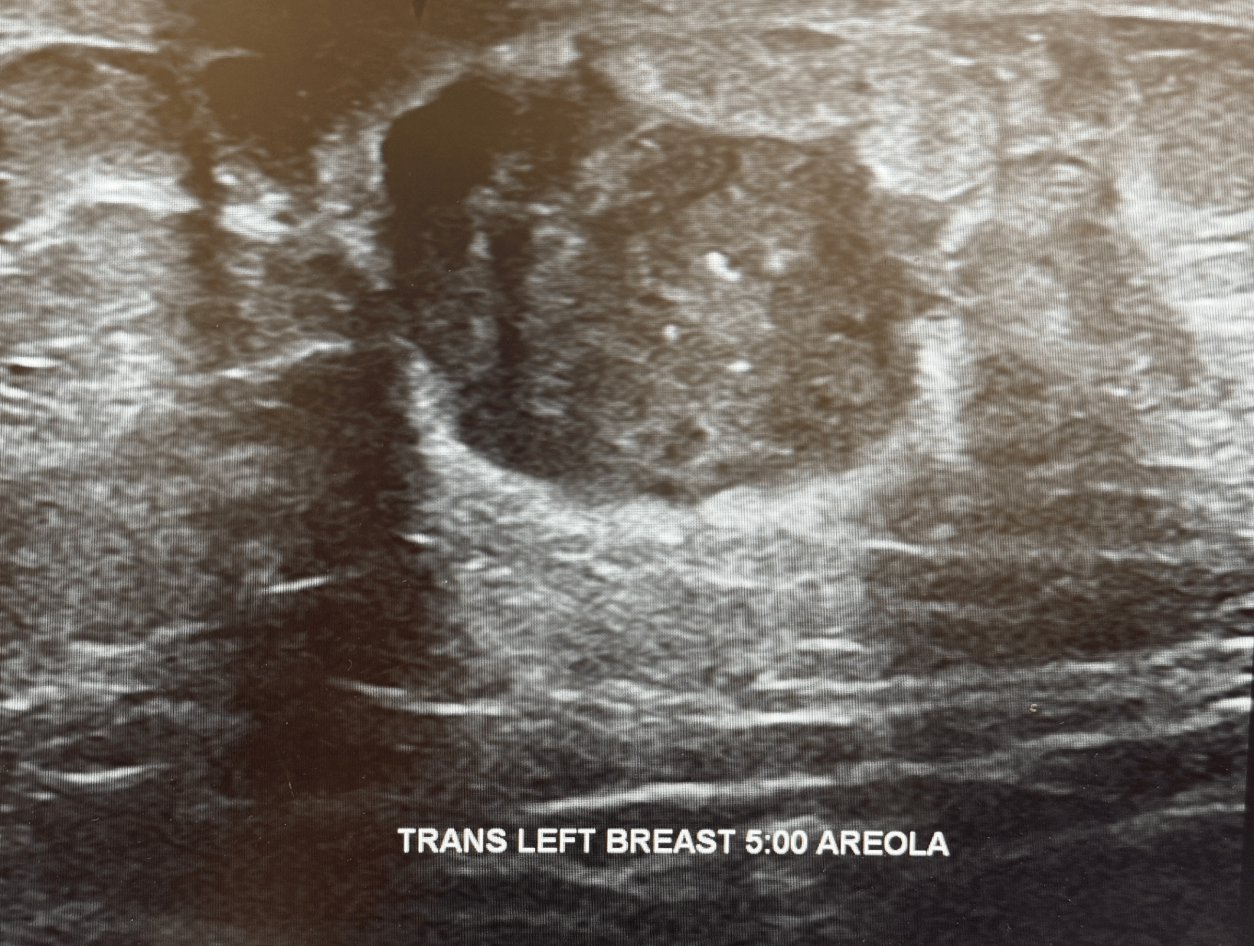
Multi-lobulated and spiculated solid vascular mass worrisome for malignancy. Consultation with a breast surgeon was recommended.

**Figure 2:**
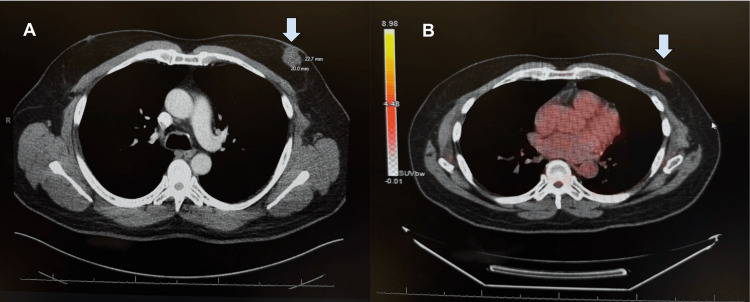
A 22 x 20 mm left breast mass suspicious for malignancy (arrow) with mild left axillary lymphadenopathy. A: Grayscale version of CT image; B: Heatmap version of CT image

**Figure 3:**
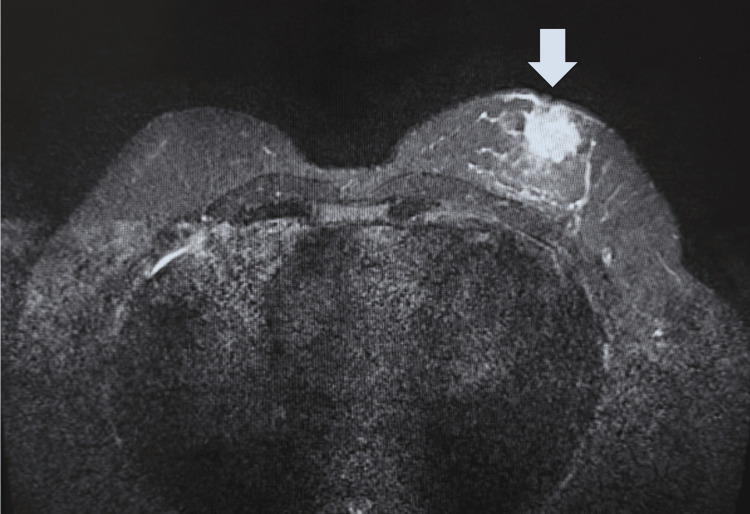
MRI showing a 2.7 x 2.3 x 2.4 cm irregularly shaped enhancing mass (arrow) of the left retroareolar region with rapid uptake, correlating to the biopsy-proven malignancy.

A 57-year-old male presented to the emergency department complaining of a lump and pain in his breast in February of 2024. Initial ultrasound revealed a multi-lobulated and spiculated solid vascular mass worrisome for malignancy (Figure 1). A subsequent CT scan of the chest with contrast demonstrated a 20 x 23 mm left breast mass and mild left axillary lymphadenopathy (Figure 2). At this time, the radiologist also noted seeing a nonspecific left adrenal nodule and recommended a further workup with MRI after the breast biopsy was completed. In April 2024, the patient underwent an ultrasound-guided left breast and axillary biopsy. The pathology report showed a 1.4 cm grade 2 invasive ductal cell carcinoma. The left axillary node biopsy was positive for metastatic carcinoma. He subsequently underwent an abdominal and breast MRI. The breast MRI depicted that the breast tissue was composed of scattered fibroglandular tissue and showed a 2.7 x 2.3 x 2.4 cm irregularly shaped enhancing mass of the left retroareolar region with rapid uptake, correlating to the biopsy-proven malignancy (Figure 3). His abdominal MRI revealed the previously discovered adrenal nodule, which showed insignificant changes since its initial discovery. However, the nodule could not be confirmed as an adenoma based on MRI criteria. The patient underwent a PET scan in May 2024. Findings included a 3 cm hypermetabolic soft tissue mass in the retroareolar region of the left breast, with a standardized uptake value (SUV) of 11, and a 2.3 cm hypermetabolic left adrenal nodule with an SUV of 5.3, concerning for distant metastasis. At this time, the patient was put on an individual chemotherapy regimen consisting of doxorubicin and cyclophosphamide q14 days x four cycles followed by paclitaxel 80 mg/m² weekly x 12 weeks. Pegfilgrastim 6 mg once per cycle was also administered subcutaneously. He was seen by the oncologist periodically for evaluations, and repeat PET and MRI evaluations were scheduled to be completed after the patient finished his chemotherapy regimen in November of 2024. The patient was subsequently put on 20 mg of tamoxifen by mouth once a day. 

**Figure 4:**
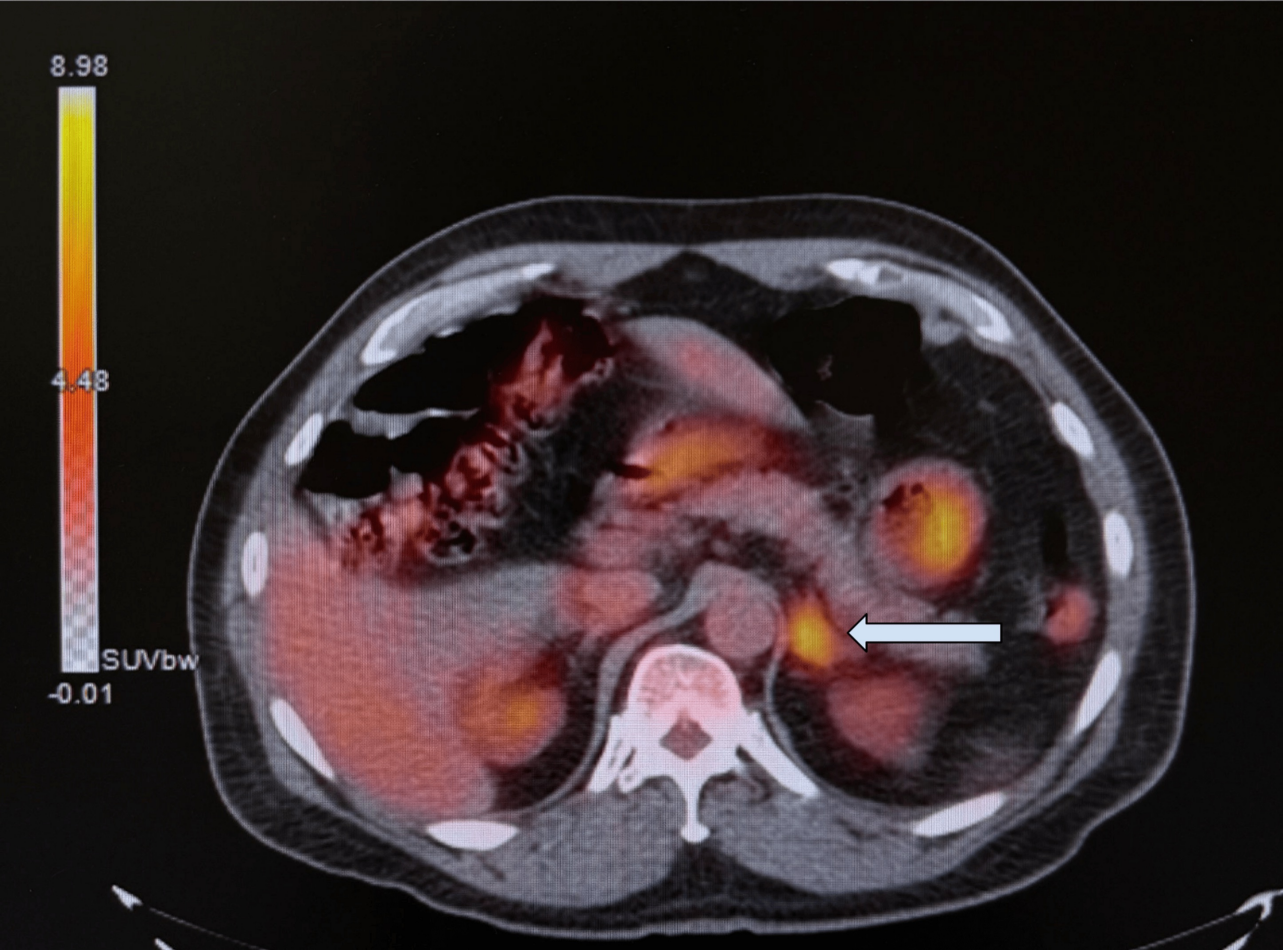
PET scan demonstrating nodular density with hypermetabolic activity of the left adrenal gland (arrow).

**Figure 5:**
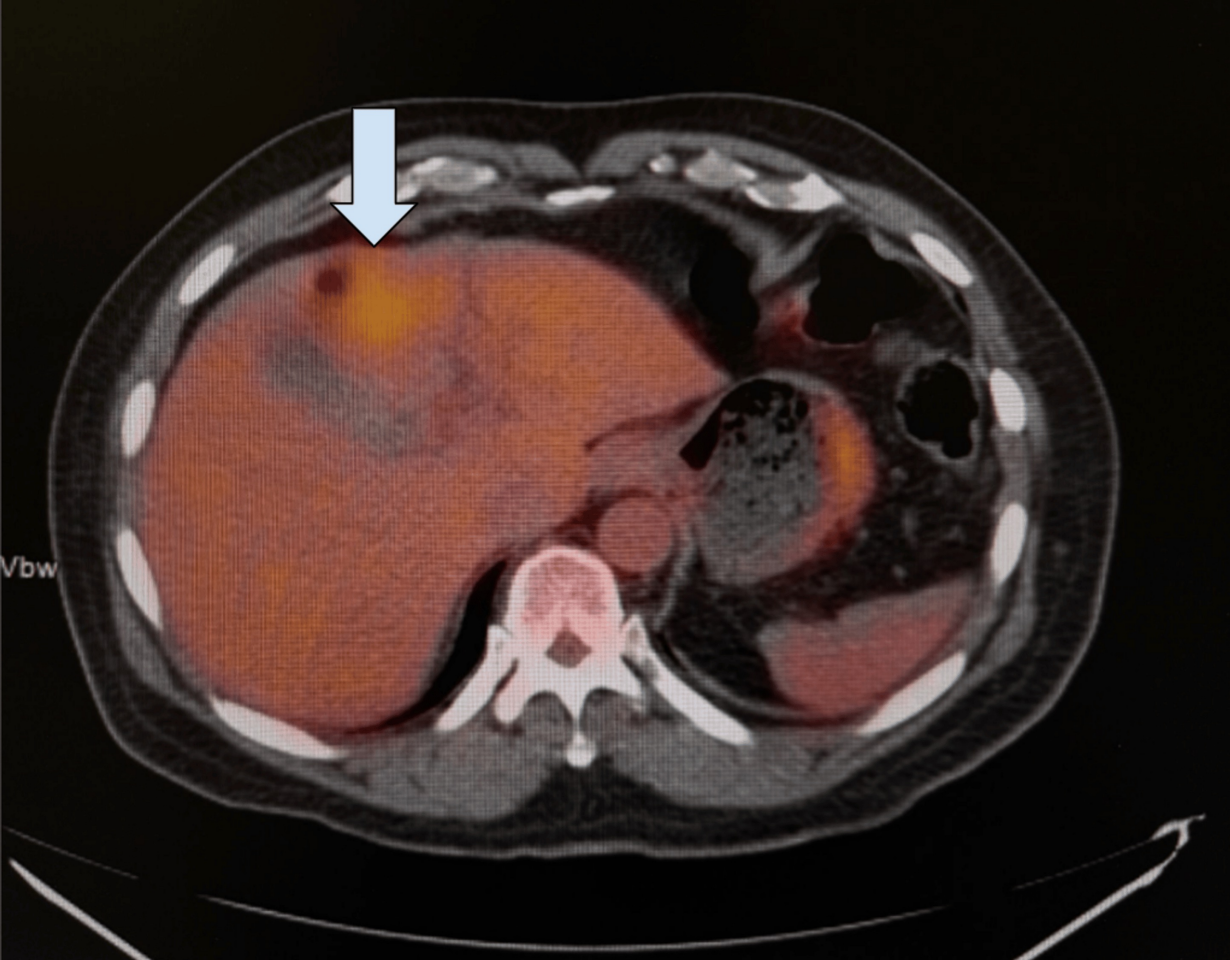
PET scan demonstrating focal increased uptake in the inferior segment of the liver, close to the hepatic flexure, with an SUV of 5.8 (arrow). SUV: standardized uptake value

An additional PET scan was completed in December 2024 as part of a comprehensive clinical evaluation. Although the radiology report noted a significantly decreased size of the retroareolar density with only a low level of metabolic activity, serving as evidence of treatment response, the nodular enlargement of the left adrenal gland with continued hypermetabolic activity was still evident (Figure 4). Additionally, a focal increased uptake in the inferior segment of the liver, close to the hepatic flexure, with an SUV of 5.8 was also seen (Figure 5). General surgery was consulted at this time and planned for a triple-phase CT after the patient’s mastectomy.

He underwent a radical left mastectomy, a left sentinel node biopsy, and a simple right mastectomy in January of 2025. The diagnosis in this male patient, based on final pathology, ultimately was a T1N1M0, estrogen receptor-positive (ER+), progesterone receptor-positive (PR+), and human epidermal growth factor receptor 2-negative (HER2-), corresponding to American Joint Committee on Cancer Stage IIA (AJCC Stage IIA) invasive ductal cell carcinoma of the left breast. The patient was put on leuprolide postoperatively. Findings of the triple-phase CT revealed no suspicious hepatic lesions and an unchanged adrenal nodule characterized as either metastatic disease or a lipid-poor adenoma. Laboratory evaluation demonstrated plasma fractionated catecholamines and metanephrines that were within normal limits, effectively ruling out a pheochromocytoma. Although plasma renin was slightly elevated, serum aldosterone levels were within normal limits, making primary aldosteronism less likely. For this reason, evaluative suppression studies were not indicated. The repeated suspicious PET and MRI imaging evaluations, combined with the inconclusive laboratory evaluation, led the oncologist and general surgeon to believe that an adrenalectomy was likely the best course of treatment. The plan was to complete the procedure after he completed his course of radiotherapy.

**Figure 6:**
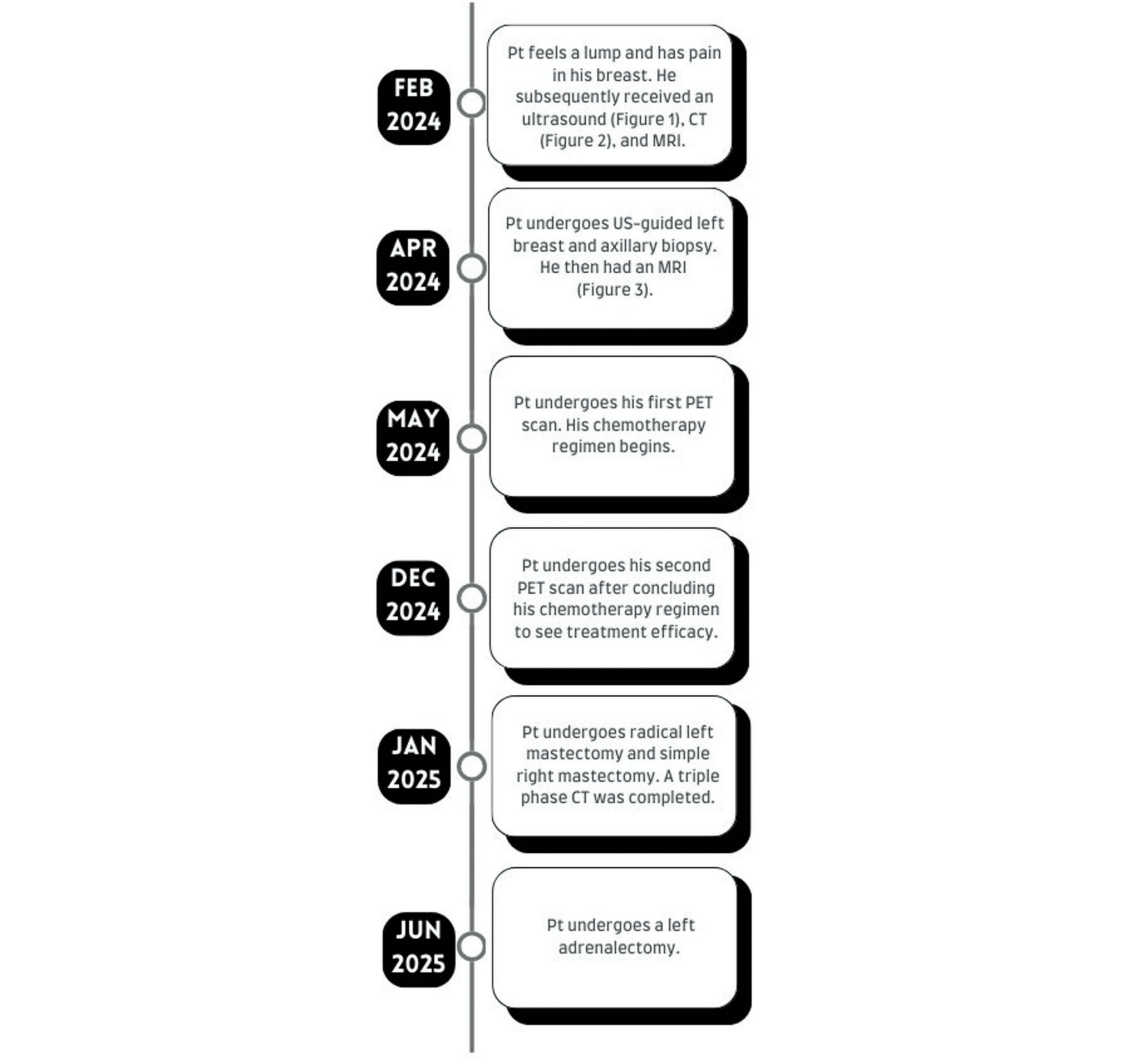
Timeline of the patient's imaging workup throughout the duration of treatment.

The patient underwent a left adrenalectomy in June of 2025. Intraoperative frozen pathology of a 3 x 2.7 x 0.6 cm portion of the left adrenal gland showed a benign collection of adrenal and fibroadipose tissue. Permanent postoperative pathology of the entire gland demonstrated, too, an aggregate of adrenal and fibroadipose tissue, consistent with a benign adrenal adenoma. After an extensive diagnostic workup that lasted over a year, the patient was now free of any lesions, both benign and malignant (Figure 6). The patient successfully recovered and was discharged with orders to follow up in six weeks. 

## Discussion

This case demonstrates the potential diagnostic challenges that may arise when pursuing a clinical care plan for suspected metastatic disease, especially when the condition is rare. Although CT and PET imaging were extremely valuable tools in the decision-making process, they were insufficient in differentiating between benign and malignant pathology. It is generally agreed upon that adrenal masses should be removed without acquiring a biopsy first, especially if the patient has a prior history of an extra-adrenal malignancy [9]. This is because of increased risks of perioperative hemorrhage and secondary hematomas, while conferring no additional therapeutic effect or decreased risk of mass recurrence [10]. According to the American Association of Endocrine Surgeons, if an adrenal incidentaloma is discovered and is suspicious for sole metastatic disease while appearing resectable in an optimal candidate, then resection, rather than biopsy, may hold a benefit [11]. Taking a multifaceted approach, the surgeon and patient ultimately agreed that a left adrenalectomy was the most suitable course of action. While this case affirms the importance of a meticulous diagnostic workup, it also calls attention to the need for balancing surgical intervention with the uncertainty of imaging. In the presence of an ambiguous initial tissue pathology, surgical teams must weigh the possibility of cautionary overtreatment against the risk of missing a solitary metastatic deposit that could alter prognostic outcomes. This case exemplifies the interplay between vigilance and intervention, demonstrating the integral value in integrating radiologic, surgical, clinical, and oncologic perspectives in the operative decision-making process [12]. Ultimately, no other sites of metastatic disease were identified, and the patient had a favorable response to both the primary breast cancer treatment and the robotic-assisted adrenalectomy that followed.

The rare incidence of male breast cancer may, too, contribute to significant uncertainties in clinical guidelines, particularly in cases involving disease progression or suspected metastasis. Most guidelines are derived from studies in females, despite potential differences in neoplastic biology, hormonal responsivity, and the overall course of disease progression [13]. This case highlights how such gaps could potentially influence clinical decision-making, especially when managing nonspecific findings in patient populations that are unfamiliar. The absence of sex-specific data may leave physicians relying on generally accepted cancer treatment protocols, which may not always necessarily apply across sexes. 

## Conclusions

This case highlights the nuanced complexities when managing incidental adrenal lesions in patients with rare malignancies such as male breast cancer. Although imaging findings may raise concern for metastatic disease, especially when a patient has a history of malignancy and no imaging findings to suggest otherwise, they are not absolute. Contingent upon the clinical context and available tools, surgical intervention should be considered. In this case, the adrenal mass ultimately was diagnosed as a benign fibroadenoma. As male breast cancer remains seldom represented in research, situations like this one provide valuable insight into optimizing the diagnostic workup and subsequent treatment guidance within such a unique patient population.
